# In-hospital Outcomes of Pediatric Cardiac Surgery Admissions for Acute Infective Endocarditis in Congenital Heart Disease

**DOI:** 10.1007/s00246-025-04037-7

**Published:** 2025-09-26

**Authors:** Mohamed F. Elsisy, Tammy Ruth, Garrett Coyan, Daniel E. Dulek, David Parra

**Affiliations:** 1 Division of Pediatric Cardiology, Department of Pediatrics, Monroe Carell Jr. Children’s Hospital at Vanderbilt, Vanderbilt University Medical Center, Nashville, TN USA; 2 Division of Pediatric Cardiac Surgery, Department of Cardiac Surgery, Vanderbilt University Medical Center, 2200 Children’s Way, Nashville, TN 37232 USA; 3 Division of Pediatric Infectious Diseases, Department of Pediatrics, Vanderbilt University Medical Center, Nashville, TN USA

**Keywords:** Infective endocarditis, Congenital heart disease, Morbidity, Mortality

## Abstract

Infective endocarditis (IE) is uncommon in children with congenital heart disease (CHD), and limited data exist regarding in-hospital outcomes of those requiring cardiac surgery. We retrospectively reviewed hospitalizations from the pediatric health information system from January 2016 to November 2024. Among 357,031 unique pediatric patients with CHD (≤ 21 years), 2108 patients (0.5%) were hospitalized for IE. The index hospitalizations during which cardiac surgery was performed were included. We aimed to characterize in-hospital outcomes among CHD children undergoing cardiac surgery for IE, with sub-analysis of those who required surgery versus medical therapy. The incidence of IE in CHD was 5.6 cases per 1000. Microorganism coding was unavailable in 41.3%; *Staphylococcus* (24.1%) and *Streptococcus* (22.3%) were the most common. Stroke occurred in 10.7%. Prosthetic valve endocarditis was present in 9.4%. Overall, 30% of patients required cardiac surgery, most commonly pulmonary valve or conduit procedures (36.6%) and tricuspid valve surgery (16.7%). Two-valve surgery was performed in 13.3%. In-hospital mortality was comparable between surgical and medical patients (10.1% vs 9.6%, *p* = 0.7). Permanent pacemaker implantation was more common after surgery (6.6% vs 2.2%, *p* < 0.01). Length of hospital stay and hospitalization costs were higher in the surgical group. On multivariable analysis, older age, heart failure, stroke, and extracorporeal membrane oxygenation (ECMO) were associated with in-hospital mortality. IE in children with CHD remains associated with substantial morbidity and mortality. In-hospital mortality remains high despite cardiac surgery. Older age, heart failure, stroke, and ECMO requirement are independent factors of in-hospital mortality.

**Supplementary Information:**

The online version contains supplementary material available at 10.1007/s00246-025-04037-7.

## Background

Infective endocarditis (IE) is a rare condition in children with congenital heart disease (CHD), with substantial morbidity and mortality [1, 2]. Children with CHD are at higher risk for developing IE, with a risk of 15–140 times higher than the general population [3, 4]. The cumulative incidence of IE in CHD patients varies considerably in the literature, ranging from 4.1 to 11.1 per 10,000 person-years [5, 6]. IE accounts for 0.1 to 0.8 per 1000 admissions for pediatric patients in other reports [7, 8], with increasing incidence due to the improved survival in patients with CHD [9]. IE in patients with CHD usually results from the presence of foreign cardiac prosthetic material, implantable cardiac devices, residual shunts, endothelial shear stress, and exposure to healthcare procedures during hospitalizations [1].

Cardiac surgery is often necessary in patients admitted with IE [10–12]. Surgery for IE in children with CHD is challenging due to the complexity of their heart anatomy and hemodynamics, previous cardiac surgeries and procedures, the presence of prosthetic materials (patches, valves or conduits) and the small body size for a prosthetic valve if indicated [10]. In contrast to CHD-associated IE in adults, data regarding outcomes of surgical treatment for IE in pediatric patients with underlying CHD is lacking. We therefore utilized the Pediatric Health Information System (PHIS) database to assess clinical characteristics and in-hospital outcomes in these patients from a national database perspective.

## Methods

### Dataset

The Pediatric Health Information System (PHIS) database was utilized to obtain our target population (https://www.childrenshospitals.org/). The PHIS database is an administrative dataset that contains clinical and resource utilization data from approximately 50 tertiary care children’s hospitals in the USA. Participating hospitals, including our institution (Monroe Carell Jr. Children’s Hospital at Vanderbilt) are affiliated with the Children’s Hospital Association (CHA) (Lenexa, KS, USA). The PHIS data provides patient-level information such as demographics, admission data, diagnoses, procedures, length of hospital stay, and cost of hospitalization. These data are tested for quality control measures before inclusion.

Our study was exempted by the Vanderbilt University Institutional Review Board (IRB) in agreement with the common rule (45 CFR 46.104 (d) category (4) (ii)).

### Patient Population

The PHIS database was queried from January 2016 to November 2024 for inpatient hospitalizations for pediatric patients ≤ 21 years of age with an *International Classification of Disease, 10th Revision* (ICD-10) code of infective endocarditis (I33.0, I33, I38, I33.9, I38X, I39.0, I39.1, I39.2, I39.3, I39.4, I39.8, B37.6, T82.6). Patients with diagnosis codes indicating the presence of CHD were included (Supplementary file, Table 1). PHIS database is encounter-based, so we limited our analysis to the index hospitalization for IE during which cardiac surgery was performed. This ensured that each individual patient was counted once for the purposes of surgical outcomes, avoiding duplications from readmissions.

Hospitalization for scheduled/elective cardiac surgery was only included in the analysis if preceded by an initial hospitalization for IE at the same hospital, thereby minimizing double-counting (*n* = 43 out of 632). Our study followed a similar approach to data extraction and cohort selection to ensure comparability with a similar prior study [13].

Patients with chronic medical conditions including oncologic, significant gastrointestinal, end-stage renal disease, and prematurity (< 32-week gestation) were excluded. These excluded conditions were identified based on ICD-10 codes (Supplementary Data*)*.

Baseline demographics included year of index hospitalization, age, sex, ethnicity, and geographic region. Comorbidities were identified based on ICD-10 diagnosis codes. Microbiology data were also obtained. ICD-10 and Current Procedural Terminology (CPT) codes were utilized to identify cardiac surgeries performed during hospitalization.

### Statistical Analysis

All statistical analyses were performed using IBM SPSS Statistics version 28 (IBM Corporation, Armonk, NY). Continuous variables were presented as medians with interquartile ranges (IQR) based on the data distribution, and comparisons were performed using the non-parametric Wilcoxon rank-sum test. Categorical parameters were reported as frequencies and percentages, and variables were compared using the Chi-square test or Fisher’s exact test (if the number of the variable is less than 5). A multivariable logistic regression model was created to identify factors significantly associated with in-hospital mortality. The Risk Adjustment for Congenital Heart Surgery (RACHS-2) category was included as a fixed covariate to adjust for procedural complexity, allowing estimation of associations between other clinical factors and in-hospital mortality independent of baseline surgical risk. Covariates chosen for inclusion in the regression model were selected according to clinical relevance and prior studies. No formal stepwise, forward, or backward selection algorithms were applied. These variables included age at index surgery, surgical complexity, staphylococcal infection, heart failure, prosthetic IE, stroke, type of CHD, multivalve surgery, requirement of ECMO, and pacemaker implantation. Odds ratios (OR) with 95% confidence intervals (CI) were reported. *p* < 0.05 was considered statistically significant.

## Results

### Baseline Characteristics

Patients’ characteristics and CHD diagnoses are summarized in Table 1.

**Table 1:** Demographics and baseline characteristics in children with congenital heart disease undergoing cardiac surgery for infective endocarditis

	Non-surgical treatment (*n* = 1476)	Surgical group (*n* = 632)	*p* value
Age in years (median [IQR]	2.7 [0.04–11.8]	9 [0.7–14.8]	< 0.01
Age groups			
0–1	513 (34.8%)	163 (25.8%)	< 0.01
1–3	133 (9%)	35 (5.5%)	0.007
3–6	114 (7.7%)	29 (4.6%)	0.009
6–9	89 (6%)	63 (10%)	0.001
9–21	419 (28.4%)	285 (45.1%)	< 0.01
Sex			0.051
Male	825 (55.9%)	383 (60.6%)	
Female	651 (44.1%)	249 (39.4%)	
Ethnicity			
White	900 (61%)	397 (60%)	0.66
African American	253 (17.1%)	100 (15.8%)	0.56
Hispanic (missing, *n* = 76)	161 (11.3%)	52 (8.6%)	0.07
Other	122 (8.6%)	74 (12.3%)	0.009
Geographic region			< 0.01
Midwest	345 (23.4%)	163 (25.8%)	
Northeast	202 (13.7%)	105 (16.6%)	
South	616 (41.7%)	218 (34.5%)	
West	313 (21.2%)	146 (23.1%)	
Diagnoses			
Right-sided heart lesions	454 (30.8%)	201 (31.8%)	0.63
Left-sided heart lesions	549 (37.2%)	278 (44%)	0.003
Septal defects	916 (62.1%)	371 (58.7%)	0.14
Conotruncal malformation	451 (30.6%)	227 (35.9%)	0.01
Urgent or emergent admission status	1173 (79.5%)	439 (69.5%)	< 0.01
Comorbidities			
Heart failure	342 (23.2%)	263 (41.6%)	< 0.01
Prosthetic valve endocarditis	90 (6.1%)	108 (17.1%)	< 0.01
Embolic Stroke	137 (9.3%)	89 (13.6%)	0.003
Brain mycotic aneurysm	3 (0.2%)	6 (0.9%)	0.02
Pulmonary embolism	116 (7.9%)	102 (16.1%)	< 0.01
Splenic infract	14 (0.9%)	11 (1.7%)	0.12
Renal infract	25 (1.7%)	12 (1.9%)	0.74
Musculoskeletal infections	62 (4.2%)	29 (4.6%)	0.69
IV drug user	2 (0.1%)	1 (0.2%)	1.0

*IQR* interquartile range

A total of 357031 individual pediatric patients (≤ 21 years of age) with a diagnosis of CHD (Supplementary file, Table 2) were hospitalized during the study timeframe. Of them, 2108 patients (0.5%) were hospitalized for infective endocarditis. The included patients were distributed evenly across three distinct age groups: 32.1% in infants (0–12 months), 34.5% were aged between 1 and 12 years, and 33.4% were adolescents. Male sex accounted for 57% of the entire cohort. The incidence of IE in patients with CHD has increased from 0.6% in 2016 to 0.8% in 2024. The estimated risk of IE in patients with CHD is 5.6 per 1000 cases.

A total of 632 patients (30%) underwent cardiac surgery during the index hospitalization for IE. Patients who underwent surgical treatment were older (9 years [IQR (interquartile range), 0.7–14.8] vs 2.7 years [IQR, 0.04–11.8], *p* < 0.01). The presence of heart failure, stroke, brain mycotic aneurysm, and pulmonary embolism was more common in the surgical group (*p* < 0.05). Prosthetic valve infective endocarditis (PIE) was more frequently observed in the surgical group (108 patients (17.1%) vs 90 patients (6.1%), *p* < 0.01). Among those who required surgical intervention, the existing prosthetic valve location was pulmonary valve or conduit in 64 patients, aortic valve in 14 patients, mitral valve in 14 patients, tricuspid valve in 4 patients, and other non-valvar locations in 8 patients.

### Microbiology Data

Microbiology information regarding the causative organism is depicted in Table 2. A code for any microorganism was not reported in 41.3% of patients. Staphylococcus was the most reported bacteria in 507 patients (24.1%) followed by streptococcus species in 471 patients (22.3%). Distribution was similar in the two groups.

**Table 2:** Microbiology data

	Medical treatment (*n* = 1476)	Surgical group (*n* = 632)	*p* value
Staphylococcus	351 (23.8%)	156 (24.7%)	0.66
Streptococcus	328 (22.2%)	143 (22.6%)	0.84
*H. influenza*	14 (0.9%)	10 (1.6%)	0.21
*E. coli*	57 (3.9%)	15 (2.4%)	0.09
Klebsiella	54 (3.7%)	22 (3.5%)	0.84
Pseudomonas	42 (2.8%)	15 (2.4%)	0.54
Candida	125 (8.5%)	47 (7.4%)	0.43

### Performed Surgeries

Previous cardiac surgery was present in 371 patients (58.7%). Detailed surgical interventions are reported in Table 3. Single valve surgery was required in the majority of patients (517 patients, 81.8%). Isolated pulmonary valve surgery (repair or replacement) or right ventricle to pulmonary trunk conduit replacement was the most common surgery performed (231 patients, 36.6%). Isolated tricuspid valve surgery was the second most common intervention in 109 patients (16.7%). Type of valve intervention, whether it is repair or replacement, was provided in the Supplementary file Table 3*.*

**Table 3:** Surgical data of children with IE undergoing cardiac surgery

Surgery performed	*N* = 632
Single valve surgery	517 (81.8%)
Isolated mitral valve surgery (repair or replacement)	99 (15.7%)
Isolated aortic valve surgery	73 (11.6%)
Isolated pulmonary valve or conduit surgery	231 (36.6%)
Isolated tricuspid valve surgery	109 (16.7%)
Multi-valve surgery	84 (13.3%)
Concomitant aortic and mitral valves surgery	7 (1.1%)
Concomitant mitral and tricuspid valves surgery	26 (4.1%)
Concomitant tricuspid and pulmonary valve or conduit surgery	25 (4.5%)
Unspecified valve surgery	62 (9.8%)
Other non-valvar cardiac surgery	31 (4.9%)
Redo heart surgery	371 (58.7%)

Classifications of cardiac lesions with corresponding surgical interventions are depicted in Table 4. Mitral and aortic valve procedures were performed in approximately 50% of patients with underlying left-sided lesions; interestingly, 37.8% of patients required pulmonary valve/conduit interventions. Pulmonary valve/conduit interventions were the predominant operation in right-sided and conotruncal heart lesions. Tricuspid valve procedures were more common in septal lesions.

**Table 4:** Classifications of cardiac lesions and corresponding surgical interventions

Left-sided lesions	
Mitral valve procedures	69 (24.8%)
Aortic valve procedures	62 (22.3%)
Tricuspid valve procedures	69 (24.8%)
Pulmonary valve/conduit procedures	105 (37.8%)
Right-sided lesions	
Mitral valve procedures	28 (13.9%)
Aortic valve procedures	16 (8%)
Tricuspid valve procedures	66 (32.8%)
Pulmonary valve/conduit procedures	109 (54.2%)
Conotruncal lesions	
Mitral valve procedures	19 (8.4%)
Aortic valve procedures	35 (15.4%)
Tricuspid valve procedures	35 (15/4%)
Pulmonary valve/conduit procedures	137 (60.4%)
Septal lesions	
Mitral valve procedures	102 (27.5%)
Aortic valve procedures	48 (12.9%)
Tricuspid valve procedures	138 (37.2%)
Pulmonary valve/conduit procedures	117 (31.5%)

Classifications of RACHS scores according to type of cardiac lesion and type of surgical intervention are detailed in Tables 5 and 6.

**Table 5:** Classification of RACHS-2 score according to CHD Type

	Left-sided lesions (*n* = 278)	Right-sided lesions (*n* = 201)	Conotruncal lesions (*n* = 227)	Septal lesions (*n* = 371)
Missing	3 (1.1%)	2 (1%)	3 (1.3%)	4 (1.1%)
Category 1	33 (11.9%)	26 (12.9%)	26 (11.5%)	64 (17.3%)
Category 2	60 (21.6%)	58 (28.9%)	84 (37%)	105 (28.3%)
Category 3	74 (26.6%)	37 (18.4%)	34 (25%)	80 (21.6%)
Category 4	28 (10.1%)	27 (13.4%)	42 (18.5%)	47 (12.7%)
Category 5	52 (18.7%)	30 (14.9%)	15 (6.6%)	36 (9.7%)

**Table 6:** Classification of RACHS-2 score according to type of surgical intervention

	Mitral valve procedures (*n* = 136)	Tricuspid valve procedures (*n* = 170)	Aortic valve procedures (*n* = 100)	Pulmonary valve/conduit procedures (*n* = 263)
Missing	0	1 (0.6%)	1 (1%)	2 (0.8%)
Category 1	2 (1.5%)	44 (25.9%)	24 (24%)	40 (15.2%)
Category 2	53 (39%)	43 (25.3%)	16 (16%)	91 (34.6%)
Category 3	54 (39.7%)	33 (19.4%)	39 (39%)	39 (14.8%)
Category 4	13 (9.6%)	14 (8.2%)	10 (10%)	31 (11.8%)
Category 5	3 (2.2%)	19 (11.2%)	6 (6%)	28 (10.6%)

### In-hospital Outcomes

In-hospital mortality was comparable between the two groups. The requirement of extracorporeal membrane oxygenation (ECMO) was similar in both groups. Permanent pacemaker implantation was more common in the surgical group (6.6% vs 2.2%, *p* < 0.01). The length of hospital stay was longer in the surgical patients (33 days [IQR, 15–74] vs 26 days [8–76], *p* = 0.025). The estimated cost of hospitalization was significantly more expensive in the surgical group (244934 US$ [126094–609873] vs 133302 US$ [IQR, 37684–460001], *p* < 0.01). In-hospital outcomes are depicted in Table 7.

**Table 7:** In-hospital outcomes of children undergoing cardiac surgery for IE

	Medical treatment (*n* = 1476)	Surgical group (*n* = 632)	*p* value
In-hospital mortality	142 (9.6%)	64 (10.1%)	0.72
ECMO	88 (6%)	47 (7.4%)	0.21
Permanent pacemaker	32 (2.2%)	42 (6.6%)	< 0.01
Length of hospital stay	26 [8–76]	33 [15–74]	0.025
Estimated cost of hospitalization	133,302 [37684–460001]	244,934 [126094–609873]	< 0.01

*ECMO* extracorporeal membrane oxygenation

### Multivariable Analysis of Factors Associated with In-hospital Mortality

A multivariable logistic regression model was performed to identify factors associated with in-hospital mortality and thee results are presented in Table 8. Variables included in the model were selected based on clinical relevance and prior literature. Age was significantly associated with mortality, with younger patients demonstrating lower odds of death (OR = 0.9 per year decrease, 95% CI [0.88–0.98], *p* ≤ 0.01).

**Table 8:** Multivariable analysis for factors associated with in-hospital mortality

Factors	OR	95% CI	*p* value
Age in years	0.93	0.88–0.98	< 0.01
Staphylococcus infection	0.86	0.51–1.40	0.51
RACHS score	1.55	1.27–1.89	< 0.01
Requirement of cardiac surgery	1.46	0.90–2.3	0.12
Heart failure	1.74	1.12–2.71	0.014
Prosthetic IE	1.64	0.18–1.85	0.21
Embolic Stroke	2.6	1.5–4.5	< 0.01
Underlying left-sided lesions	1.46	0.90–2.4	0.14
Underlying right-sided lesions	0.53	0.33–0.86	0.009
Underlying septal defects	1.38	0.83–2.28	0.21
Underlying conotruncal lesions	0.88	055–1.42	0.61
Multi-valve surgery	0.57	0.18–1.85	0.35
ECMO	5.44	3.21–9.20	< 0.01
PPM	1.77	0.85–3.7	0.13

*OR* odds ratio, *RACHS_2 score* risk adjustment for congenital heart surgery score, *ECMO* extracorporeal membrane oxygenation, *PPM* permanent pacemaker

ECMO requirement was significantly associated with in-hospital mortality after accounting for potential confounders (OR = 5.4, 95% CI [3.2–9.2], *p* < 0.01). Presence of heart failure was a strong risk factor for in-hospital death (OR = 1.7, 95% CI [1.1–2.7], *p* = 0.01). Lastly, the occurrence of stroke was also predictive of mortality (OR = 2.6, 95%CI [1.5–4.5], *p* < 0.01). Patients with underlying right-sided cardiac lesions were inversely related to in-hospital mortality (OR = 0.5, 95% CI [0.3–0.8], *p* = 0.009).

## Discussion

### The Incidence of IE in Pediatric Patients with CHD

Our data demonstrate that the estimated incidence of IE in hospitalized children with CHD is 5.6 per 1000 unique cases, substantially higher than in the general pediatric population [7]. Our finding aligns with a population-based study from the Quebec CHD database from 1988 to 2010 [14] which reported an incidence of 6.1 cases per 1000 children (birth—18 years of age). Another population-based analysis from Taiwan from 1997 to 2005 found that the overall incidence of IE in children with CHD (*n* = 24729) who were followed until 2010 was 11.13 per 10000 person-years [5]. The rate of IE in adults with CHD (ACHD) is greater than that in children with CHD. A nationwide study from the Netherlands [15] demonstrated that the overall incidence of IE in ACHD patients is 1.33 cases per 1000 person-years, approximately 3 times higher than the reported incidence in children from the Quebec CHD database (0.41 cases/1000 person-years). The authors explained the overall difference by cardiac lesion distribution and prevalence of prosthetic materials, with left-sided CHD lesions being more common in ACHD patients compared to the pediatric cohort. Additionally, adult patients are more vulnerable to developing IE due to the greater complexity of their cardiac disease and higher incidence of comorbidities and acquired diseases [16].

### Age-Related Patterns and Potential Risk Factors

In accordance with previously published reports [9, 17, 18], in our cohort, the distribution of IE was relatively even across the three age groups: infants (0–1 year), middle childhood (1–9 years) and adolescents (9–21 years). Infants and young children are at higher risk for prolonged hospitalization in neonatal and pediatric intensive care units, high rates of indwelling venous catheters, and other invasive procedures, which can increase the incidence of healthcare-associated infective endocarditis. On the other hand, adolescents with CHD are more likely to experience IE for various reasons, including a higher prevalence of cardiac prosthesis, presence of residual shunts, and behavioral risk factors, including intravenous illicit drug use, body piercing or tattoos under non-sterile conditions, and undergoing dental procedures without prophylaxis. Prior literature corroborates these associations, emphasizing the importance of targeted education and preventive care in adolescents with CHD [19, 20].

### Surgical Interventions and Clinical Complexity

Approximately, 30% with CHD and IE in our cohort underwent cardiac surgery. This surgery frequency is similar to the frequency of surgery in a prior cohort of IE in pediatric patients not limited to patients with pre-existing CHD [9] that reported an incidence of 33.8% of pediatric patients with pre-existing heart disease admitted for IE; however, it was unclear whether the cardiac surgery occurred before or after the IE diagnosis. Literature reported an incidence ranging between 25 and 50% in the general population [10, 11]. Pulmonary valve replacement or right ventricle to pulmonary artery conduit replacement was the most common performed surgery in our cohort. These conduits are usually homografts containing valves and are performed in cyanotic lesions including tetralogy of Fallot with pulmonary atresia. These conduits tend to degenerate and constrict over time, resulting in stenosis and regurgitation, which create a high-risk environment for seeding bacteria. A prior study suggests that different types and materials of RV-PA conduits may be associated with a higher risk of IE, such as bovine jugular grafts that were associated with a substantially higher risk of late IE compared to other valved conduits [21]. Surgery for pulmonary valve and/or right ventricular outflow tract (RVOT) IE ranges from 14% to 28.3% in single-center experiences [18, 22, 23]. In accordance with a large prospective cohort in the adult population [24], our data demonstrate that prosthetic valve endocarditis was present in 9.4% of patients. Interestingly, we did not observe any difference in in-hospital mortality between prosthetic valve endocarditis and those with native endocarditis, suggesting that modern perioperative care and the emergence of dedicated pediatric cardiac intensive care units may mitigate some excess risk. Prosthetic valve IE was significantly associated with death, demonstrated by a retrospective study from a tertiary center in Australia [23].

### In-hospital Outcomes and Healthcare Resource Utilization

The overall in-hospital mortality in our investigation was 9.8% in the whole cohort, falling within the range reported in other pediatric IE studies (2.1%–15%) [10, 18, 23, 25]. Notably, our results reveal no difference in in-hospital mortality between those who required cardiac surgery and those who were managed medically. This is consistent with findings from large retrospective cohorts and supports the notion that surgical intervention in pediatric IE, when indicated, does not inherently confer increased perioperative risk [22, 25, 26].Our cohort also illustrates substantial utilization of healthcare resources in the surgical group. The surgical patients were sicker, with higher rates of heart failure, septic emboli, and postoperative permanent pacemaker implantation.

### Predictors of In-hospital Mortality

In agreement with the literature, multivariable analysis identified that increasing age was associated with higher in-hospital mortality. This can be possibly explained by the higher complexity of long-standing CHD, presence of multiple prior surgeries, ventricular dysfunction, and pulmonary hypertension. Heart failure was common in our cohort with an incidence of 28.7% and was predictive of in-hospital mortality. Heart failure can result from valve regurgitation, particularly aortic valve, and is considered an indication for cardiac surgery according to the AHA guidelines [27]. Embolic stroke was significantly associated with in-hospital mortality on multivariable analysis. Embolic events are common complications of IE with an incidence between 30 and 40% of left-sided IE [28, 29]. Maraey et al. [30]queried the National Inpatient Database from 2016 to 2020 for IE patients with and without embolic stroke. They concluded that embolic stroke is significantly associated with lower survival rates, prolonged hospitalization, and healthcare resource utilization.

In conclusion, this study reveals several significant results regarding the characteristics and outcomes of IE in CHD patients. Despite still being a rare complication, IE occurs with an estimated incidence of 5.6 per 1000 hospitalized patients with CHD. IE most commonly occurs in two age groups—infants and adolescents—suggesting age-related risk factors.

Cardiac surgery is required in nearly one-third of IE patients, with replacement of the pulmonary valve or right ventricle to pulmonary artery conduit and tricuspid valve surgery being the most common procedures. Despite the substantial operative risk, in-hospital mortality was not significantly different between operated patients and nonoperated counterparts.

However, patients who underwent surgery had more complex clinical courses, including increased rates of postoperative permanent pacemaker implantation, increased duration of hospitalization, and increased health care utilization. Notably, several factors including older age at the diagnosis of IE, stroke, and ECMO requirement were significantly associated with in-hospital mortality and are useful benchmarks for risk stratification.

### Limitations

Our study is limited by the inherent factors in a large retrospective database. PHIS database is an administrative database and is subject to miscoding [31]. However, the CHA follows strict quality measures in order to minimize errors in coding and data entry from the participating hospitals. Our data is obtained from 50 tertiary children’s hospitals in the USA, which limited the generalizability of our results. Comorbidities, clinical diagnoses, and index procedures were identified using ICD-10 and CPT codes; hence, granular results regarding severity of disease, echocardiographic data, and more details about the index procedures are lacking. A key limitation of PHIS database is the inability to determine the temporal sequence of events relative to index surgery, as PHIS database is encounter based and does not include timestamps for discharge diagnoses. It is not possible to distinguish whether certain variables such as stroke occurred preoperatively or postoperatively. This limits our ability to accurately identify preoperative risk factors and may introduce misclassification bias. Our analysis also includes the hospitalization during which cardiac surgery was performed and does not capture the medical-only admissions for patients who may have undergone staged or delayed surgical intervention after discharge. As such, our cohort may limit generalizability to all children with IE, particularly those managed medically or with planned delayed surgery.

## Conclusion

Infective endocarditis in children with CHD has substantial mortality and morbidity with higher incidence in infants and adolescents. Surgical patients demonstrate a high burden of disease with a greater need for healthcare resource utilization. While cardiac surgery is often required, it does not impact hospital mortality. Older age, embolic stroke, and heart failure were associated with worse outcomes. These findings highlight the importance of early recognition and prevention of IE in this vulnerable population.

## Supplementary Information

Below is the link to the electronic supplementary material.

Supplementary file1 (DOCX 20 kb)

## Data Availability

Data supporting our results are available. Our cohort is extracted from a publicly available database (Pediatric Health Information System [PHIS]) database. Our spreadsheet and analysis are stored in Excel files and we are willing share our analysis as requested.

## References

[1] Cahill TJ et al (2019) Contemporary epidemiology of infective endocarditis in patients with congenital heart disease: a UK prospective study. Am Heart J 215:70–77 31299559 10.1016/j.ahj.2019.05.014

[2] Cahill TJ et al (2017) Challenges in infective endocarditis. J Am Coll Cardiol 69(3):325–344 28104075 10.1016/j.jacc.2016.10.066

[3] Eleyan L et al (2021) Infective endocarditis in paediatric population. Eur J Pediatr 180:3089–3100 33852085 10.1007/s00431-021-04062-7

[4] Baltimore RS et al (2015) Infective endocarditis in childhood: 2015 update: a scientific statement from the American Heart Association. Circulation 132(15):1487–1515 26373317 10.1161/CIR.0000000000000298

[5] Sun LC et al (2017) Risk factors for infective endocarditis in children with congenital heart diseases - a nationwide population-based case control study. Int J Cardiol 248:126–130 28811093 10.1016/j.ijcard.2017.08.009

[6] Moore B et al (2017) Incidence, predictors and outcomes of infective endocarditis in a contemporary adult congenital heart disease population. Int J Cardiol 249:161–165 29121720 10.1016/j.ijcard.2017.08.035

[7] Elder RW, Baltimore RS (2015) The changing epidemiology of pediatric endocarditis. Infect Dis Clin North Am 29(3):513–524 26311357 10.1016/j.idc.2015.05.004

[8] Snygg-Martin U et al (2021) Cumulative incidence of infective endocarditis in patients with congenital heart disease: a nationwide, case-control study over nine decades. Clin Infect Dis 73(8):1469–1475 34036324 10.1093/cid/ciab478 PMC8528398

[9] Day MD et al (2009) Characteristics of children hospitalized with infective endocarditis. Circulation 119(6):865–870 19188508 10.1161/CIRCULATIONAHA.108.798751

[10] Murakami T et al (2012) Factors associated with surgery for active endocarditis in congenital heart disease. Int J Cardiol 157(1):59–62 21176981 10.1016/j.ijcard.2010.11.016

[11] Prendergast BD, Tornos P (2010) Surgery for infective endocarditis: who and when? Circulation 121(9):1141–1152 20212293 10.1161/CIRCULATIONAHA.108.773598

[12] Hoen B, Duval X (2013) Infective endocarditis. N Engl J Med 368(15):1425–1433 23574121 10.1056/NEJMcp1206782

[13] Pasquali SK et al (2012) Trends in endocarditis hospitalizations at US children’s hospitals: impact of the 2007 American Heart Association antibiotic prophylaxis guidelines. Am Heart J 163(5):894–899 22607869 10.1016/j.ahj.2012.03.002 PMC3408007

[14] Rushani D et al (2013) Infective endocarditis in children with congenital heart disease. Circulation 128(13):1412–1419 24060942 10.1161/CIRCULATIONAHA.113.001827

[15] van Melle JP et al (2023) Infective endocarditis in adult patients with congenital heart disease. Int J Cardiol 370:178–185 36273665 10.1016/j.ijcard.2022.10.136

[16] Nakagawa N (2022) Infective endocarditis in congenital heart disease. Endocarditis-diagnosis and treatment. IntechOpen, London

[17] Sélégny M et al (2021) Infective endocarditis in children: a 10-year multicentric study. Arch Cardiovasc Dis Suppl 13(4):300–301

[18] Russell HM et al (2013) Outcomes of surgical therapy for infective endocarditis in a pediatric population: a 21-year review. Ann Thorac Surg 96(1):171–175 23602067 10.1016/j.athoracsur.2013.02.031

[19] Armstrong ML, DeBoer S, Cetta F (2008) Infective endocarditis after body art: a review of the literature and concerns. J Adolesc Health 43(3):217–225 18710675 10.1016/j.jadohealth.2008.02.008

[20] Carvajal V et al (2024) Endocarditis in adult congenital heart disease patients: prevention, recognition, and management. Curr Cardiol Rep 26(9):1031–1045 39212775 10.1007/s11886-024-02103-9 PMC11379749

[21] Mery CM et al (2016) Risk factors for development of endocarditis and reintervention in patients undergoing right ventricle to pulmonary artery valved conduit placement. J Thorac Cardiovasc Surg 151(2):432-9-441.e1-2 26670191 10.1016/j.jtcvs.2015.10.069

[22] Carrillo SA et al (2023) Surgical outcomes of infective endocarditis in pediatrics: moving the needle to a contemporary, multidisciplinary approach. J Thorac Cardiovasc Surg 165(1):275–284 35537892 10.1016/j.jtcvs.2022.03.031

[23] Khoo B et al (2019) Outcomes of surgery for infective endocarditis in children: a 30-year experience. J Thorac Cardiovasc Surg 158(5):1399–1409 31383559 10.1016/j.jtcvs.2019.06.024

[24] Murdoch DR et al (2009) Clinical presentation, etiology, and outcome of infective endocarditis in the 21st century: the International Collaboration on Endocarditis-Prospective Cohort Study. Arch Intern Med 169(5):463–473 19273776 10.1001/archinternmed.2008.603 PMC3625651

[25] Shamszad P et al (2013) Early surgical therapy of infective endocarditis in children: a 15-year experience. J Thorac Cardiovasc Surg 146(3):506–511 23312102 10.1016/j.jtcvs.2012.12.001

[26] Nomura F et al (1995) Surgical intervention for infective endocarditis in infancy and childhood. Ann Thorac Surg 60(1):90–95 7598627

[27] Baddour LM et al (2015) Infective endocarditis in adults: diagnosis, antimicrobial therapy, and management of complications. Circulation 132(15):1435–1486 26373316 10.1161/CIR.0000000000000296

[28] Steckelberg JM et al (1991) Emboli in infective endocarditis: the prognostic value of echocardiography. Ann Intern Med 114(8):635–640 2003709 10.7326/0003-4819-114-8-635

[29] Vilacosta I et al (2002) Risk of embolization after institution of antibiotic therapy for infective endocarditis. J Am Coll Cardiol 39(9):1489–1495 11985912 10.1016/s0735-1097(02)01790-4

[30] Maraey A et al (2023) Abstract 11693: comparison of in-hospital outcomes in infective endocarditis patients with and without embolic stroke. Circulation 148(Suppl_1):A11693–A11693

[31] Strickland MJ et al (2008) The importance of nomenclature for congenital cardiac disease: implications for research and evaluation. Cardiol Young 18(S2):92–100 19063779 10.1017/S1047951108002515 PMC3743224

