# Supplementary material for: In-hospital Outcomes of Pediatric Cardiac Surgery Admissions for Acute Infective Endocarditis in Congenital Heart Disease
Source: Pediatr Cardiol. 2025 Sep 26;47(6):2607–14. doi: 10.1007/s00246-025-04037-7 (PMC13498656; doi:10.1007/s00246-025-04037-7)
Supplement: Supplementary file 1 [file 246_2025_4037_MOESM1_ESM.docx]

**Table 1 ICD 10 Codes Utilized to Identify Diagnoses and Procedures**

| Endocarditis | - I330 Acute and subacute infective endocarditis - I33 Acute and subacute endocarditis - I38 Endocarditis, valve unspecified - I339 Acute endocarditis, unspecified - I38X Endocarditis, valve unspecified - I390 Endocarditis and mitral valve disorders in disease classified elsewhere - I391 Endocarditis and aortic valve disorders in disease classified elsewhere - I392 Endocarditis and tricuspid valve disorders in disease classified elsewhere - I393 Endocarditis and pulmonary valve disorders in disease classified elsewhere - I394 Endocarditis and multiple valve disorders in disease classified elsewhere - I398 Endocarditis, valve unspecified in disease classified elsewhere - B376 Candidal endocarditis - T826 Infection and inflammatory reaction due to cardiac valve prosthesis |
| --- | --- |
| Congenital Heart Disease | - Q200 Common arterial trunk - Q203 Discordant ventriculoarterial connection - Q201 DORV - Q205 Discordant atrioventricular connection - Q208 Other congenital malformations of heart chambers and connections - Q213 TOF - Q204 DILV - Q210 VSD - Q211 ASD - Q212 AVSD - Q214 Aortopulmonary septal defect - Q218 Other congenital malformation of cardiac septa - Q219 Congenital malformation of cardiac septum, unspecified - Q223 Other congenital malformations of pulmonary valve - Q220 Pulmonary valve atresia - Q221 Congenital pulmonary valve stenosis - Q222 Congenital pulmonary valve insufficiency - Q224 Congenital tricuspid stenosis - Q226 Hypoplastic right heart - Q228 Other congenital malformation of tricuspid valve - Q225 Ebstein’s anomaly - Q230 Congenital aortic valve stenosis - Q231 Congenital aortic valve insufficiency - Q232 Congenital mitral valve stenosis - Q233 Congenital mitral valve insufficiency - Q234 HLHS - Q244 Congenital subaortic stenosis - Q242 Cor triatriatum - Q243 Pulmonary infundibular stenosis - Q238 Other congenital malformations of aortic and mitral valves - Q239 Congenital malformation of aortic and mitral valves, unspecified - Q251 Coarctation of aorta - Q254 Congenital malformation of aorta - Q252 Aortic valve atresia - Q253 Supravalvular aortic stenosis - Q259 Congenital malformation of great arteries, Unspecified - Q255 Atresia of pulmonary artery - Q257 Coarctation of pulmonary artery - Q264 Anomalous pulmonary venous connection, unspecified - Q262 TAPVR - Q263 PAPVR |
| Mitral valve repair | ***Annuloplasty:**  02UG07Z Supplement Mitral Valve with Autologous Tissue Substitute, Open Approach  02UG08Z Supplement Mitral Valve with Zooplastic Tissue, Open Approach  02UG0JZ Supplement Mitral Valve with Synthetic Substitute, Open Approach  02UG0KZ Supplement Mitral Valve with Nonautologous Tissue Substitute, Open Approach  ***Other Repair:**  027G04Z Dilation of Mitral Valve with Drug-eluting Intraluminal Device, Open Approach  027G0DZ Dilation of Mitral Valve with Intraluminal Device, Open Approach  027G0ZZ Dilation of Mitral Valve, Open Approach  02NG0ZZ Release Mitral Valve, Open Approach  02QG0ZZ Repair Mitral Valve, Open Approach  02VG0ZZ Restriction of Mitral Valve, Open Approach  028D0ZZ Division of Papillary Muscle, Open Approach  02QD0ZZ Repair Papillary Muscle, Open Approach  02890ZZ Division of Chordae Tendineae, Open Approach  02Q90ZZ Repair Chordae Tendineae, Open Approach  02QG0ZZ Repair Mitral Valve, Open Approach |
| Mitral valve replacement |  |
| Aortic valve replacement | 02RD07Z, 02RF08Z, 02RF0KZ, 02RF47Z, 02RF48Z, 02RF4KZ, X2RF032, X2RF432) |
| Aortic valve repair | 02QF0ZZ, 02QF0ZJ, |
| Tricuspid valve replacement |  |
| TV repair | 02QJ0ZZ, 02QJ3ZZ, 02QJ4ZZ, 02QJ0ZG, 02QJ3ZG, 02QJ4ZG, 02UJ08Z, 02UJ0JZ, 02UJ38Z, 02UJ3JZ |
| Pulmonary valve replacement | 02RH08Z, 02RH0JZ, 02RH38Z, 02RH3JZ, 02RH07Z, 02RH0KZ, 02RH37H, 02RH37Z, 02RH38H, 02RH38L, 02RH38M, 02RH3JH, 02RH3KH, 02RH3KZ, 02RH47Z, 02RH48Z, 02RH4JZ, 02RH4KZ |
| Pulmonary valve repair | 02QH0ZZ, 02QH3ZZ, 02QH4ZZ |
| Stroke | G463, G464, I693, I6930, I69369, I639, I6340, I69365, G9511, I69959, I6931, I69359, Hemorrhage: I600, I601, I602, I603, I604, I605, I606, I607, I608, I609, I610, I611, I612, I613, I614, I615, I616, I618, I619, I620, I621, I629.  Infraction: I630, I631, I632, I633, I634, I635, I636, I638, I639  I650, I651, I652, I653, I658, I659, I660, I661, I662, I663, I664, I668, I669  Cerebral aneurysm: I671, I729 |
| Infective organism |  |
| Streptococcus | B950, B951, B952, B953, B954, B955, |
| Staph | B956, B957, B958 |
| Hemophilus influenza | B963 |
| Ecoli | B962 |
| Klebsiella | B961 |
| Pseudomonas | B965 |
| Candida | B376, B37 |
| Pulmonary embolism | I2690, I26 |
| Splenic infract/abscess | D733, D735 |
| Renal infract/abscess | N289, N151 |
| Musculoskeletal | M0080, M00859, M00879, M00819,M00829, M00829, M00849, M00869, M009, M00839, M0088, M0000, M0010, M00018, M86, M8620, M4620, M4623, M4624, M4625, M4626, M4627, M4627, M86211, M86212, M86219, M86221, M86222, M86229, M86241, M86242, M86249, M86251, M86252, M86259, M8629, M869, M4628, M86149, M86239, M86269, M86279, M86619, M86649 |

**Table.2 Classification of CHD**

| Right-sided heart lesions | Pulmonary atresia  Pulmonary stenosis  Pulmonary artery stenosis  Hypoplastic right heart syndrome  Tricuspid atresia  Ebstein’s anomaly |
| --- | --- |
| Left-sided heart lesions | Aortic stenosis  Aortic atresia  Hypoplastic left heart syndrome  Mitral stenosis  Mitral atresia  Coarctation of aorta  Hypertrophic obstructive cardiomyopathy  Subaortic stenosis |
| Septal defects | Atrial septal defects  Ventricular septal defects  Atrioventricular septal defects  Patent ductus arteriosus  Aortopulmonary window |
| Conotruncal lesions | Tetralogy of Fallot  Double outlet right ventricle  Interrupted aortic arch  Truncus arteriosus  Transposition of great arteries |

**Table 3. Type of Valve Surgical Intervention**

| Mitral valve repair | 86 (13.6% |
| --- | --- |
| Mitral valve replacement | 68 (10.8%) |
| Aortic/truncal valve replacement | 71 (11.2%) |
| Aortic valve repair | 34 (5.4%) |
| Tricuspid valve repair | 153 (24.2%) |
| Tricuspid valve replacement | 10 (1.6%) |
| Pulmonary valve repair | 26 (4.1%) |
| Pulmonary valve replacement or Right ventricle to pulmonary trunk conduit replacement | 252 (39.9%) |
